# Prevalence of Respiratory Disease and Associated Factors in an Urban Area of Delhi

**DOI:** 10.4103/0970-0218.43227

**Published:** 2008-10

**Authors:** Pragti Chhabra, Geetanjali Sharma, Anjur T Kannan

**Affiliations:** Department of Community Medicine, University College of Medical Sciences and GTB Hospital, Delhi - 110 095, India

**Keywords:** Prevalence, respiratory disease, respiratory symptoms, risk factors

## Abstract

**Objectives:**

To study the prevalence of respiratory morbidity and its associated factors in urban Delhi.

**Study Design and Setting:**

A cross-sectional, house-to-house survey was conducted in an urban upper middle class locality.

**Materials and Methods:**

All the residents aged 18 years or more were administered a questionnaire to identify the major symptoms of chronic respiratory tract disease — chronic cough, chronic phlegm, dyspnea and wheezing. The prevalence of all these symptoms in different groups was calculated. Chi square test and logistic regression were applied to determine the significant factors.

**Results:**

A total of 3465 individuals were interviewed of which 1756 (50.68%) were males and 1709 (49.3%) were females. Only 9.05% of the men smoked. The overall prevalence of chronic cough, chronic phlegm and dyspnea was 2.0%, 1.2% and 3.4%, respectively. The prevalence of wheezing was 3.2%. All the symptoms increased with age (*P* < 0.05). No significant difference was observed in these symptoms between males and females. Less educated and retired individuals were more likely to have respiratory symptoms. The prevalence of chronic cough, chronic phlegm, dyspnea and wheezing was 5.8%, 2.9%, 9.9% and 8.7%, respectively, among smokers, which was significantly higher than that observed in nonsmokers. Logistic regression analysis revealed that age and smoking remained significant factors for occurrence of all the respiratory symptoms.

## Introduction

Chronic respiratory diseases are defined as chronic diseases of the respiratory tract and other structures of the lung. The most common diseases are asthma and chronic obstructive pulmonary disease. These diseases represent a challenge to the public health in both industrialized and developing countries because of their frequency and economic impact. In 1990, the World Health Organization/World Bank Global Burden of Disease study estimated the global prevalence of chronic obstructive pulmonary disease (COPD) to be 9.33 per 1000 individuals for men and 7.33 per 1000 for women. The prevalence was observed to be higher in industrialized countries.(1–3)

In India, there have been only a few population-based studies on the prevalence of COPD,(4–6) some hospital-based studies(7) and some in specific population groups such as workers(8) or patients attendants.(9) Only few of these studies have used standardized questionnaires, operational definitions of chronic bronchitis and asthma. Few studies with regard to the risk factors associated with chronic respiratory disease in urban areas have been conducted. The present study was conducted to study the prevalence of respiratory disease and its associated factors in an urban area of Delhi.

## Materials and Methods

This was a cross-sectional study conducted in an urban colony of East Delhi. The colony comprises approximately 1100 independent houses. The Municipal Corporation of Delhi has classified different colonies into categories from A to G, based on the unit area value of the house, i.e., it is a measure of the economic status of the residents of these houses.

The colony under study has been classified into category D, which falls in the upper middle class. We obtained a sample size of 3173, assuming that the population of age 18 years and above was 3500 in this area, the prevalence of respiratory disease as 5%, allowing an error of 5% on either side and a confidence level of 99%. We decided to include all the houses in this survey. We obtained the directory of the residents from the Resident Welfare Association and performed a house-to-house survey. All the residents aged 18 years or above, staying for more than six months in the area were included in the survey. A questionnaire based on the British Medical Research Council and the American Thoracic Society questionnaires was developed.(10) It was designed to identify the following major symptoms of chronic respiratory tract disease — chronic cough, chronic phlegm, dyspnea and wheezing. If applied properly, questionnaires are a valid method of investigation in the epidemiology of respiratory diseases. The respondents were also asked whether they had been informed by a doctor that they had a chronic respiratory condition.

Definitions:*Chronic cough* was defined as cough on most days for three consecutive months or more during the year for the past two years or more. *Chronic phlegm* was defined as phlegm on most days for three consecutive months or more during the year for the past two years or more. *Dyspnea* was defined as breathlessness when walking, which required the subject to stop or slow down for breathing while walking on the level. *Wheezing* referred to the occurrence of wheezing/whistling sounds in breathing associated with breathlessness on most days or nights.

The subjects were classified according to smoking status as follows: current smokers, who smoked regularly within one month prior to the interview; nonsmokers, who never smoked or occasionally smoked; and ex-smokers, stopped smoking more than one month prior to the interview.

Analysis: the prevalence of all the symptoms with regard to age, gender, education and smoking was calculated. A chi square test was applied to analyze the difference in the prevalence in various groups. To control for confounding, multiple logistic regression was performed using the factors found to be significant on univariate analysis. Data analysis was performed using the SPSS software version 11.

## Results

There were 1100 houses of which 127 were being used for commercial activity, while 42 were found locked on two visits and residents of 31 houses were either unavailable or uncooperative. Thus, a total of residents of 943 houses were eligible for the study, and residents of 870 houses were interviewed; this yielded a response rate of 92.3%.

A total of 3465 subjects were interviewed and included in the study. Table 1 shows the demographic profile of the study population. The overall prevalence of chronic cough, chronic phlegm and dyspnea was 2.0%, 1.2% and 3.4%, respectively [Table 2]. The prevalence of wheezing was 3.2%. All the symptoms increased with age; individuals 30 years or below had the least possibility for all the symptoms, while subjects above 70 years of age were most likely to have respiratory symptoms (*P* < 0.05). No significant difference was observed in the prevalence of these symptoms amongst males and females. Individuals with primary or less education were more likely to have chronic cough, while other respiratory symptoms although higher in this group did not show any significant difference. The prevalence of all the symptoms was the highest amongst the retired individuals and the lowest among students; however, the difference between chronic cough and dyspnea was statistically significant. The occurrence of all respiratory symptoms was significantly higher in smokers as compared to nonsmokers. The categories smokers and ex-smokers were combined since the proportion of ex smokers was very low (0.7%) and a separate analysis was not possible.

**Table 1:** Demographic profile of the study population

Factor	Category	n (%of total)
Age (in years)	18–30	992 (28.63)
	31–50	1433 (41.36)
	51–70	853 (24.62)
	>70	187 (5.4)
Gender	Male	1756 (50.68)
	Female	1709 (49.32)
Education	Nil	59 (1.9)
	1–5 years	255 (7.36)
	6–12 years	635 (18.33)
	12–15 years	1940 (55.99)
	>15 years	576 (16.62)
Occupation	Student	301 (8.69)
	Service	604 (17.43)
	Business	964 (27.82)
	Retired	491 (14.17)
	Housewife	1105 (31.89)
Smoking	Smoker	158 (4.7)
	Nonsmoker	3183 (94.6)
	Ex-smoker	24 (0.7)

**Table 2:** Prevalence of respiratory symptoms in relation to factors

	Chronic cough	Chronic phlegm	Dyspnea	Wheezing
Age in years				
18–30	1.0 (10)*	0.6 (6)*	1.8 (18)*	1.8 (18)*
31–50	1.0 (15)*	0.7 (10)*	2.8 (41)*	2.2 (32)*
51–70	3.8 (31)*	2.3 (20)*	4.8 (41)*	5.4 (46)*
>70	8.1 (13)*	4.0 (7)*	11.4 (18)*	8.1 (15)*
Gender				
Male	2.0 (35)	1.4 (24)	3.5 (62)	3.5 (62)
Female	2.0 (34)	1.1 (19)	3.3 (56)	2.8 (49)
Education				
0–5	5.1 (16)*	1.8 (5)	4.4 (13)	4.8 (15)
6–12	2.4 (15)*	1.9 (13)	3.9 (25)	3.1 (20)
13–15	0.9 (18)*	0.9 (17)	3.2 (62)	2.9 (57)
>15	3.4 (20)*	1.3 (8)	3.1 (18)	3.2 (19)
Smoking				
Smoker	5.8 (11)*	2.9 (5)*	9.9 (18)*	8.7 (16)*
Nonsmoker	1.8 (58)*	1.1 (37)*	3.0 (100)*	2.8 (85)*
Occupation				
Retired	4.3(21)*	2.4 (12)*	5.9 (29)*	4.7 (23)
Housewife	2.6 (29)*	1.4 (15)*	3.4 (37)*	3.0 (34)
Student	1.6 (5)*	1.0 (3)*	3.0 (9)*	2.9 (9)
Service	1.2 (7)*	0.8 (5)*	2.1 (13)*	2.2 (13)
Business	0.7 (7)*	0.7 (7)*	3.1 (30)*	3.3 (33)
Total	2.0 (69)*	1.2 (42)*	3.4 (118)*	3.2 (111)

* *P* < 0.05

Bivariate logistic regression analysis was performed assuming the occurrence of symptom as the dependent variable and age, education and smoking as covariates [Table 3]. Age above 50 years was a significant risk factor for chronic cough, chronic phlegm, dyspnea and wheezing. Individuals between 51 and 69 years of age were nearly three times more likely to have these symptoms, while those above 70 years were six times more likely to have chronic cough, chronic phlegm and dyspnea and 4.6 times likely to have wheezing. Smokers and ex-smokers reported respiratory symptoms occurring three times more often than nonsmokers after controlling for age and education. Graduates had the least risk of chronic cough as compared to other categories; however, the occurrence of other respiratory symptoms was not significantly associated with the educational status of the subjects. It was observed that smokers had approximately three times odds of reporting all the respiratory symptoms.

**Table 3:** Adjusted odds ratio for respiratory symptoms with regard to factors

Factor	Category	Chronic cough	Chronic phlegm	Dyspnea	Wheezing
Age (years)	18–30	1	1	1	
	31–50	1.06 (0.45–2.46)	1.07 (0.38–3.02)	1.53 (0.86–2.4)	1.18 (0.64–2.15)
	51–69	3.20* (1.48–6.94)	3.55* (1.38–9.13)	2.74 (1.52–4.97)*	3.08 (1.71–5.53)*
	>70	6.80* (2.68–17.25)	6.57* (2.01–21.4)	7.33* (3.54–15.17)	4.64* (2.10–10.25)
Education (years)	>5	1	1	1	1
	6–12	0.85 (0.40–1.79)	0.80 (0.24–2.65)	0.88 (0.40–1.94)	0.93 (0.43–1.98)
	13–15	0.66 (0.32–1.33)	1.41 (0.54–3.1)	1.30 (0.68–2.41)	0.91 (0.4.1–79)
	>15	0.31 (0.15–0.60)*	0.82 (0.33–2.02)	1.16 (0.66–2.02)	1.0 (0.58–1.74)
Smoking	Absent	1	1	1	1
	Present	3.08* (1.51–6.29)	2.43* (0.93–6.35)	3.3* (1.92–5.79)	3.07* (1.72–5.48)

* *P* < 0.05; Figures in parentheses indicate 95% confidence interval of adjusted odds ratio

## Discussion

In the present study, the overall prevalence of chronic cough, chronic phlegm and dyspnea was 2.0%, 1.2% and 3.4%, respectively. In India, few population-based studies on the prevalence of COPD have been conducted; these studies have reported figures ranging from 1.4% to 9.4% in males and 1.3% to 4.9% in females.(3–6) In a review on population studies by Jindal *et al.*, a median figure of 5% for males and 2.7% for females has been estimated.(11) We observed a lower prevalence than those mentioned above since the area studied belonged to the upper middle socioeconomic status and included young adults also. When we compared our data with other studies, the findings agree with those reported in populations belonging to upper socioeconomic class. Radha *et al.*(4) observed a prevalence of 1.96 in an affluent locality of Delhi, whereas Chhabra *et al.* noted a higher prevalence of respiratory symptoms.(12) A similar lower prevalence was observed among nonsmoker males and females in studies reported from Chandigarh from both rural and urban areas and among teachers.(13,14) However, a community-based study in the rural area of Kashmir reported a considerably higher prevalence that was attributed to domestic air pollution, lower socioeconomic status, poor housing facilities and overcrowding.(4) All these factors were distinctly lacking in our study population that may explain the lower prevalence of respiratory symptoms. In an urban area of Kashmir, a prevalence of 5.7% for chronic bronchitis(5) was reported, while in a south Indian village, a prevalence of 3.3% was reported for chronic bronchitis.(15)

All the respiratory symptoms increased with age that remained a significant risk factor for the occurrence of all the respiratory symptoms. Other studies have also observed an increase in respiratory diseases with increasing age.(4,6,9)

In the study population, no significant difference was observed in the prevalence of respiratory symptoms in males and females. Most studies from India and other countries have observed a male preponderance for the occurrence of COPD. The difference is attributed to the differential rates of smoking and occupational exposure between the two genders. The proportion of smokers in the present study population was lower than that in other studies and a majority of the subjects were employed in nonindustrial jobs. These factors may explain the similar prevalence of respiratory diseases in the two genders. Studies from rural areas, especially in the colder regions of North India have reported a higher prevalence in females, which may be due to the exposure of women to domestic smoke pollution.(16)

On univariate analysis, educational status was associated with the occurrence of respiratory symptoms, but on adjusting for age and smoking, this was not an independent factor. A study from South Africa reported higher education as a protective factor for respiratory symptoms.(17) The lack of significant association between respiratory symptoms and educational status may be due to the fact that a majority (72%) of the subjects had 12 years or more of education, and the less educated individuals were the older females in the study area.

This is a cross-sectional study, and it provides a valuable summary about the burden of respiratory symptoms in this community. However, the limitation of this study is that it failed to provide any evidence to distinguish between the cause and effect; it is based on the symptoms of respiratory disease alone and no investigations were carried out.

Thus, the present study shows that the prevalence of respiratory symptoms was low in the upper socioeconomic class. The significant risk factors are age and smoking status.
